# A Case of Multiple Metastases in the Gastric Wall Due to Needle Tract Seeding after Endoscopic Ultrasound-Guided Tissue Acquisition for Pancreatic Tail Cancer

**DOI:** 10.70352/scrj.cr.25-0577

**Published:** 2026-01-16

**Authors:** Kazuhiro Takami, Kei Nakagawa, Hiroto Sakurai, Noriko Kondo, Kuniharu Yamamoto, Akinobu Koiwai, Morihisa Hirota, Masashi Katoh, Yu Katayose

**Affiliations:** 1 Division of Hepato-Biliary-Pancreatic Surgery, Tohoku Medical and Pharmaceutical University, Sendai, Miyagi, Japan; 2 Division of Gastoroenterology, Tohoku Medical and Pharmaceutical University, Sendai, Miyagi, Japan; 3 Division of Pathology, Tohoku Medical and Pharmaceutical University, Sendai, Miyagi, Japan

**Keywords:** needle tract seeding, pancreatic cancer, EUS-TA

## Abstract

**INTRODUCTION:**

Endoscopic ultrasound-guided tissue acquisition (EUS-TA) is widely used for the diagnosis of pancreatic cancer, other pancreatic tumors, and autoimmune pancreatitis. Among the adverse events, needle tract seeding (NTS) is the most concerning. NTS is a phenomenon in which tumor cells are observed at the site of biopsy needle passage. We herein report a case of multiple gastric wall recurrences resulting from NTS after laparoscopic distal pancreatectomy for pancreatic tail cancer.

**CASE PRESENTATION:**

A 71-year-old woman was diagnosed with pancreatic tail adenocarcinoma using EUS-TA and underwent laparoscopic distal pancreatectomy following neoadjuvant chemotherapy. Three and a half years postoperatively, two submucosal gastric lesions were identified and histologically confirmed as NTS. Since there was no recurrence at any site other than the site of NTS on close examination, a total gastrectomy was performed with postoperative adjuvant chemotherapy, and the patient remained recurrence-free.

**CONCLUSIONS:**

We report a case of multiple synchronous NTS after EUS-TA. EUS-TA is a very useful examination method for pancreatic tumors; however, it should be performed with particular care in cases of invasive pancreatic ductal carcinoma after distal pancreatectomy. If NTS in the gastric wall is detected at an early stage, surgical resection is possible and the patient prognosis can be improved.

## Abbreviations

CEA
: carcinoembryonic antigen
EUS-TA
: EUS-guided tissue acquisition
FDG-PET-CT
: fluorodeoxyglucose-PET-CT
NAC
: neoadjuvant chemotherapy
SUVmax
: standardized uptake value maximum

## INTRODUCTION

Endoscopic ultrasound-guided tissue acquisition (EUS-TA) is widely performed as an essential technique for the diagnosis of pancreatic cancer, other pancreatic tumors, and autoimmune pancreatitis.^1–3)^ EUS-TA may cause complications such as bleeding, acute pancreatitis, and infection at a rate of approximately 1.7%–2.5%, but serious complications are reported to occur in approximately 1% of cases.^4,5)^ Among the adverse events reported with this procedure, needle tract seeding (NTS) is of the greatest concern because it may affect the disease prognosis. NTS is a phenomenon in which tumor cells are observed at the site of biopsy needle passage. It is a subtype of peritoneal dissemination. In this report, we present a case of multiple gastric wall recurrences resulting from NTS after laparoscopic distal pancreatectomy for pancreatic tail cancer.

## CASE PRESENTATION

**Fig. 1:**
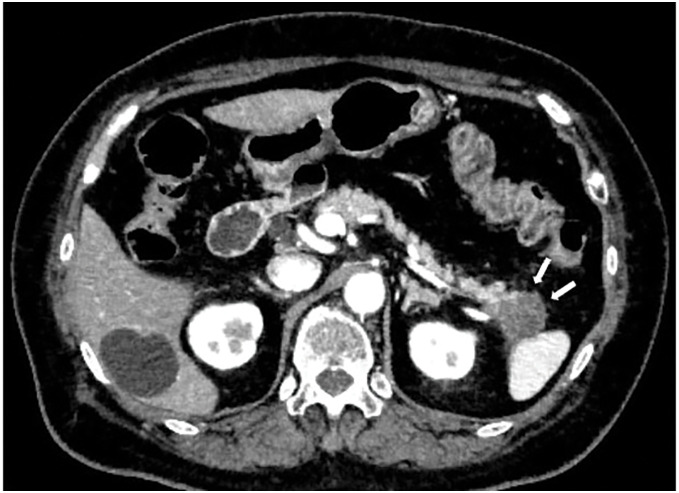
Contrast-enhanced CT showed a hypovascular tumor, 32 mm in diameter, in the pancreatic tail.

**Fig. 2:**
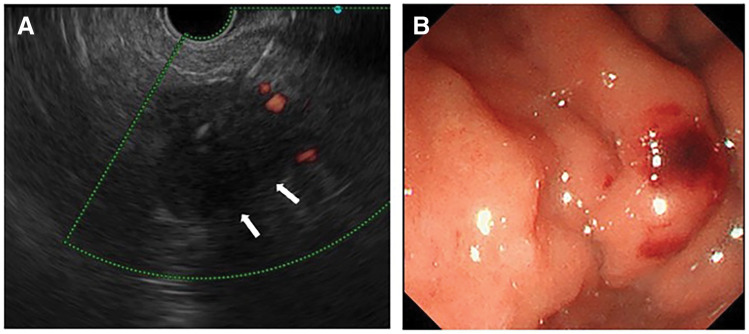
EUS-TA using a 25-gauge needle with a side hole was performed (**A**) through the posterior wall of the gastric body (**B**).

**Fig. 3:**
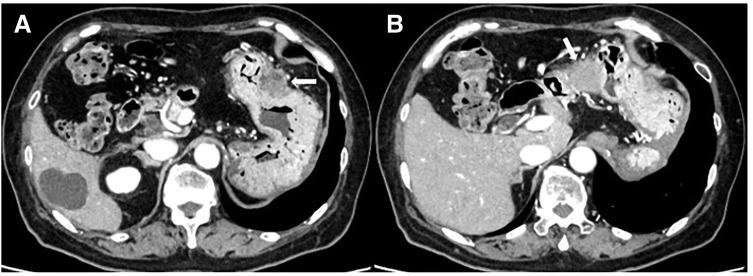
Three years and 6 months after initial pancreatectomy, CT revealed two low-density masses (**A**, **B**) in the wall of the stomach.

**Fig. 4:**
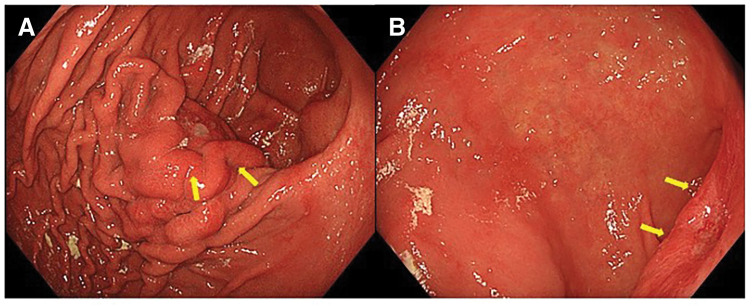
An upper gastrointestinal endoscopy revealed submucosal tumor-like lesions on the posterior wall of the gastric body (**A**) and on the lesser curvature of the antrum (**B**).

**Fig. 5:**
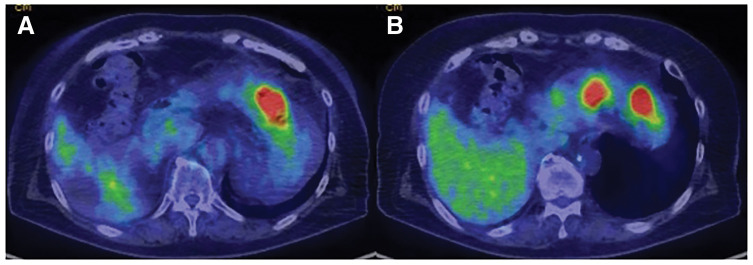
FDG-PET-CT revealed abnormal accumulation in the stomach body (SUVmax 14.2) (**A**) and the antrum (SUVmax 9.8) (**B**), but not in other areas. FDG-PET-CT, fluorodeoxyglucose-PET-CT

**Fig. 6:**
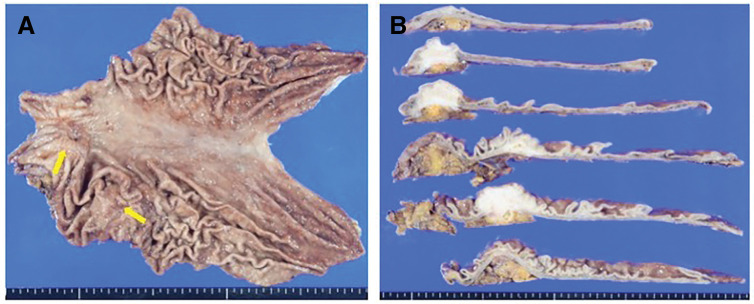
The resected specimen showed two grossly elevated lesions of 35 and 40 mm in size (**A**, **B**).

**Fig. 7:**
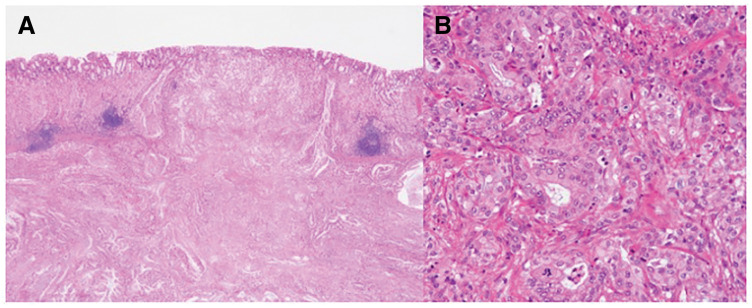
Histopathological examination of the resected stomach revealed a similar histology in the two lesions, with atypical cells with nuclear staining and enlargement proliferating in the submucosal to subserosal layers with fibrotic stromal reaction (**A**, **B**). The final pathological diagnosis was well to moderately differentiated adenocarcinoma, which was similar to those of the previously resected primary cancer in the pancreatic tail, indicating that the tumor in the stomach was recurrence-derived from needle tract seeding from pancreatic cancer.

Enhanced CT performed for close examination after a medical checkup revealed a 32 mm hypovascular mass in the pancreatic tail in a 71‑year‑old woman (**Fig. 1**). EUS-TA with fine needle was performed in two sessions of 20 strokes with a 25-gauge puncture needle (**Fig. 2A**, **2B**). Histopathological examination of the biopsy specimen revealed adenocarcinoma, which was diagnosed as a carcinoma of the pancreatic tail. After two courses of neoadjuvant chemotherapy with gemcitabine and S-1, laparoscopic distal pancreatectomy was performed. Intraoperative findings revealed no dissemination within the peritoneal cavity. The omental bursa contained only physiological adhesions between the gastric wall and transverse colon mesentery, with no adhesions observed between the stomach and the pancreas. Histopathological examination of the resected specimen revealed invasive ductal carcinoma pT3, TS2 (30 mm), INFβ, ly2, v2, ne2, mpd0, pN1b (5/39), cM0, and pStage IIB. The patient underwent adjuvant therapy with S-1 for 6 months. Surveillance was then conducted on the patient thereafter; however, at 3 years and 6 months after the surgery, the CEA level was slightly elevated at 6.2 ng/mL, and a low-density mass appeared on the gastric wall on a CT scan (**Fig. 3A**, **3B**). Upper gastrointestinal endoscopy revealed submucosal tumor-like lesions on the posterior wall of the gastric body and the lesser curvature of the antrum (**Fig. 4A**, **4B**). A biopsy confirmed adenocarcinoma in each lesion, which was considered an NTS during EUS-TA. A colonoscopy revealed no evidence of malignancy. CT showed no distant metastases, including liver or lung metastases, local recurrence, or lymph node metastases other than this lesion. FDG-PET/CT revealed abnormal tracer accumulation in the stomach body (SUVmax 14.2) and gastric antrum (SUVmax 9.8); however, there was no accumulation in other regions (**Fig. 5A**, **5B**). The patient underwent robot-assisted laparoscopic total gastrectomy for gastric wall recurrence. The resected specimen showed two grossly elevated lesions of 35 and 40 mm in size, with a white split surface (**Fig. 6A**, **6B**). Histopathological examination of the resected stomach revealed that the two lesions showed a similar histology, with atypical cells with nuclear staining and enlargement proliferating in the submucosal to subserosal layers with a fibrotic stromal reaction (**Fig. 7A**, **7B**). Immunohistochemistry showed CK7+, CK20-, MUC1+, MUC5AC+ at both sites of gastric wall recurrence. This pattern was entirely consistent with the previous pancreatic cancer immunohistochemistry. Both morphologically and based on the immunohistochemistry results, the final pathological diagnosis was well-to-moderately differentiated adenocarcinoma, which was considered consistent with seeding from pre-existing pancreatic cancer. The patient received postoperative adjuvant chemotherapy with S-1 for 6 months. The patient is currently alive with no recurrence.

## DISCUSSION

EUS-TA has become an essential technique for the definitive diagnosis of pancreatic cancer, the differential diagnosis of other pancreatic tumors, and the diagnosis of autoimmune pancreatitis, and the positive diagnosis rate for these diseases is reported to be very high.^1–3)^ In addition, the adverse event rate for this procedure is generally low at 1.7%–2.5%.^4,5)^ Therefore, it is now widely used and has become a standard procedure. Adverse events such as bleeding, pancreatitis, peritonitis, pancreatic fistula, NTS, endoscopic perforation, and bile leakage may also be problematic in such cases.^4)^

NTS is a phenomenon in which tumor cells are found in the puncture route during needle biopsy and is defined as a histopathologically proven recurrent tumor of the gastric wall after radical surgical resection.^4)^ It is considered a subtype of peritoneal dissemination of digestive system malignancy and a complication that cannot be ignored as a form of malignant disease recurrence. While complications of EUS-TA, such as bleeding and pancreatitis, are indirectly related to the long-term outcomes of patients, NTS is an adverse event that directly affects the prognosis.

The study led by the Japan Pancreatic Association in 2022 is the largest and most evidence-based report on the frequency of NTS after EUS-TA.^6)^ This study included 12109 patients who underwent surgical resection after EUS-TA, and reported the incidence of NTS in pancreatic ductal adenocarcinomas and other pancreatic tumors. The overall incidence of NTS in this study was 0.330% and 0.409% in the pancreatic ductal adenocarcinoma group and 0.071% in the other pancreatic tumor group, respectively. In this study, 38 patients with NTS were analyzed. The median time from EUS-TA to the diagnosis of NTS was 19.3 months, the median tumor size was 22 mm, and surgical resection was performed in 25 cases. In addition, it has been reported that patients who underwent gastrectomy for NTS had significantly longer survival than those who did not undergo gastrectomy. In this report, the authors also divided the patients into two groups: those who underwent transgastric EUS-TA and those who underwent transduodenal EUS-TA and reported that there was no incidence of NTS in the transduodenal EUS-TA group. This may be because the tumors punctured by transduodenal EUS-TA were localized in the pancreatic head region. Pancreaticoduodenectomy was selected as the technique for resection of the primary tumor, and the puncture route was included in the resection specimen. Although this study did not mention surgical procedures for primary lesions, if only cases with pancreatic ductal adenocarcinoma and distal pancreatectomy as the surgical procedure had been evaluated, the incidence of NTS would have been much higher. By contrast, Yane et al. reported that NTS was found in 3.4% of patients with invasive pancreatic duct cancer who underwent distal pancreatectomy after EUS-TA, which is more frequent than in previous reports.^7)^

In the present case, synchronous lesions and multiple NTS were observed in the posterior wall of the gastric body and the lesser curvature of the pyloric region. Multiple cases of NTS are rare, with only one such case reported to date.^8)^ In our case, two sessions of 20-stroke puncture sessions with a 25-gauge needle were performed during EUS-TA, and the puncture site was a single site on the posterior wall of the gastric body. The lesser curvature of the antrum cannot be a puncture site and the occurrence of NTS at this site is a mystery. Levy et al. reported that 3 of 26 patients with pancreatic cancer had malignant tumors in the gastrointestinal luminal fluid after EUS-TA.^9)^ On the other hand, Katanuma et al. considered that the histological findings at the site of gastric wall recurrence indicated that the tissue repair process after puncture is related to tumor engraftment.^10)^ Although the endoscope is reversed and puncture is performed during EUS-TA, it may be possible that contact injury occurred in the antrum area and seeding was completed during tissue repair in the same area. Additionally, mucosal damage or ulcers during the healing process may increase the risk of seeding. This case suggests that NTS may occur in areas other than puncture sites. In this case, we conducted thorough preoperative discussions and planned and performed a total gastrectomy. The primary reason was the unexplained presence of disseminated lesions at two sites. This suggested the possibility of additional lesions beyond these two recurrence sites. Furthermore, there was no evidence supporting the omission of lymph node dissection (including resection of the left gastric artery) in the area where the recurrent lesions were present. Considering the vascular supply of the stomach after distal pancreatectomy, total gastrectomy was deemed necessary. Neoadjuvant chemotherapy (NAC) may reduce NTS. However, in this case, both NAC and adjuvant chemotherapy were administered and NTS was still present. Currently, there are no established preventive procedures for NTS after EUS-TA. Therefore, at this point, we should assume a puncture route for pancreatic tumors, and if that route is not included in the primary tumor resection specimen, other modalities such as pancreatic juice cytology via an endoscopic nasopancreatic drainage tube should be considered. In addition, the possibility of NTS should always be kept in mind during surveillance of pancreatic body and tail cancers that have undergone EUS-TA, and upper gastrointestinal endoscopy should be performed routinely in addition to CT.

The prognosis of pancreatic cancer is expected to improve in the future as neoadjuvant chemotherapy becomes the standard of care, the completion rate of adjuvant chemotherapy improves with the widespread introduction of minimally invasive surgery, and the impact of NTS on the prognosis of pancreatic cancer will become more difficult to ignore.

## CONCLUSIONS

EUS-TA is a highly useful diagnostic method for pancreatic tumors. In cases in which distal pancreatectomy is planned, special attention must be paid to the puncture technique and postoperative observation because the puncture site remains after surgery. If NTS in the gastric wall is detected at an early stage, surgical resection is possible and the patient prognosis can be improved. In cases treated with EUS-TA, it is important to confirm the upper gastrointestinal tract endoscopy regularly, not only with CT, to avoid missing the optimal timing for treatment.

## DECLARATIONS

### Availability of data and materials

All data generated or analyzed during this study are included in this published article.
